# Pulmonary Neoplasms in Patients with Birt-Hogg-Dubé Syndrome: Histopathological Features and Genetic and Somatic Events

**DOI:** 10.1371/journal.pone.0151476

**Published:** 2016-03-14

**Authors:** Mitsuko Furuya, Reiko Tanaka, Koji Okudela, Satoko Nakamura, Hiromu Yoshioka, Toyonori Tsuzuki, Ryo Shibuya, Kazuhiro Yatera, Hiroki Shirasaki, Yoshiko Sudo, Naoko Kimura, Kazuaki Yamada, Shugo Uematsu, Toshiaki Kunimura, Ikuma Kato, Yukio Nakatani

**Affiliations:** 1 Department of Molecular Pathology, Yokohama City University Graduate School of Medicine, Yokohama, Japan; 2 Medical Mycology Research Center, Chiba University, Chiba, Japan; 3 Department of Pathology, Yokohama City University Graduate School of Medicine, Yokohama, Japan; 4 Department of Pathology, Kagawa Prefectural Central Hospital, Kagawa, Japan; 5 Department of Thoracic Surgery, Japanese Red Cross Nagoya Daini Hospital, Nagoya, Japan; 6 Department of Pathology, Japanese Red Cross Nagoya Daini Hospital, Nagoya, Japan; 7 Department of Respiratory Medicine, University of Occupational and Environmental Health, Japan, Kita-Kyushu, Japan; 8 Pathology and Oncology, University of Occupational and Environmental Health, Japan, Kita-Kyushu, Japan; 9 Department of Internal Medicine, Saiseikai Fukui Hospital, Fukui, Japan; 10 Department of Pathology, Saiseikai Fukui Hospital, Fukui, Japan; 11 Department of Thoracic Surgery, National Disaster Medicine Center, Tokyo, Japan; 12 Department of Pathology, National Disaster Medicine Center, Tokyo, Japan; 13 Department of Thoracic Surgery, Showa University Northern Yokohama Hospital, Yokohama, Japan; 14 Department Pathology, Showa University Northern Yokohama Hospital, Yokohama, Japan; 15 Department of Diagnostic Pathology, Chiba University Graduate School of Medicine, Chiba, Japan; Queen Mary Hospital, HONG KONG

## Abstract

Birt-Hogg-Dubé syndrome (BHD) is an inherited disorder caused by genetic mutations in the folliculin (*FLCN*) gene. Individuals with BHD have multiple pulmonary cysts and are at a high risk for developing renal cell carcinomas (RCCs). Currently, little information is available about whether pulmonary cysts are absolutely benign or if the lungs are at an increased risk for developing neoplasms. Herein, we describe 14 pulmonary neoplastic lesions in 7 patients with BHD. All patients were confirmed to have germline *FLCN* mutations. Neoplasm histologies included adenocarcinoma *in situ* (n = 2), minimally invasive adenocarcinoma (n = 1), papillary adenocarcinoma (n = 1), micropapillary adenocarcinoma (n = 1), atypical adenomatous hyperplasia (n = 8), and micronodular pneumocyte hyperplasia (MPH)-like lesion (n = 1). Five of the six adenocarcinoma/MPH-like lesions (83.3%) demonstrated a loss of heterozygosity (LOH) of *FLCN*. All of these lesions lacked mutant alleles and preserved wild-type alleles. Three invasive adenocarcinomas possessed additional somatic events: 2 had a somatic mutation in the epidermal growth factor receptor gene (*EGFR*) and another had a somatic mutation in *KRAS*. Immunohistochemical analysis revealed that most of the lesions were immunostained for phospho-mammalian target of rapamycin (p-mTOR) and phospho-S6. Collective data indicated that pulmonary neoplasms of peripheral adenocarcinomatous lineage in BHD patients frequently exhibit LOH of *FLCN* with mTOR pathway signaling. Additional driver gene mutations were detected only in invasive cases, suggesting that *FLCN* LOH may be an underlying abnormality that cooperates with major driver gene mutations in the progression of pulmonary adenocarcinomas in BHD patients.

## Introduction

Birt-Hogg-Dubé syndrome (BHD), also called Hornstein-Knickenberg syndrome, is an inherited disorder characterized by multiple pulmonary cysts and repeated pneumothorax [1–3]. Genetic mutation of the folliculin (*FLCN*) gene, which maps to chromosomal region 17p11.2, is responsible for this disorder [4]. FLCN forms a complex with FLCN-interacting protein 1 (FNIP1) and FNIP2, and the complex cross-talks with 5’-AMP-activated protein kinase (AMPK) and the mammalian target of rapamycin (mTOR) [5–8]. The principal role of FLCN is tumor suppression. In addition, recent studies have revealed the importance of FLCN in muscle homeostasis, hematopoiesis, and autophagy [9, 10].

Little attention has been paid to the cancers other than renal cell carcinoma (RCC) that occur in 20–30% of BHD patients. Most individuals with BHD have multiple pulmonary cysts; the cyst-lining cells look absolutely benign, and are generally flattened or exfoliated [11, 12]. Notably, the inner surfaces of the cysts are occasionally lined by plump pneumocytes with proliferative activity [11]. Patients with BHD frequently have episodes of recurrent pneumothoraces, indicating that the cyst walls are ruptured due to either mild enlargement pressure or tissue remodeling. We previously used immunohistochemistry to demonstrate that the pneumocytes lining the cyst wall frequently express phospho-mTOR (p-mTOR) and phospho-S6 (p-S6), supporting the hypothesis that a subset of the lining cells active mTOR signaling [11, 13]. We therefore speculated that the lung might possibly be susceptible to BHD-associated neoplasms. Some clinical studies have reported the occurrence of lung adenocarcinoma or atypical alveolar hyperplasia (AAH) in patients with BHD [14–16]. Two of 11 *Flcn* heterozygous mice had glandular neoplasms in the lung [17].

In the present investigation, we studied 14 lung neoplasms in patients with BHD who underwent video-assisted thoracoscopic surgery (VATS). All 14 lesions showed abnormal proliferation of pneumocytes and were pathologically diagnosed as adenocarcinomas, AAHs, or a micronodular pneumocyte hyperplasia (MPH) -like lesion. Histological and immunohistochemical analyses with antibodies against FLCN, mTOR pathway molecules, ALK, ROS1, and mutant/deleted forms of epidermal growth factor receptor (EGFR) were performed. Somatic mutation analyses of *FLCN*, *EGFR*, and *KRAS* were also done in microdissected neoplasms. We observed frequent loss of heterozygosity (LOH) of *FLCN* in the pulmonary neoplastic lesions of BHD patients.

## Materials and Methods

### Samples

Seven Japanese patients were enrolled in this study. Written informed consents for gene analyses were obtained from all patients. Among them, 2 patients were siblings whose clinical findings were reported previously [15]. For 1 patient who died before the study initiation, informed consent was obtained from his sister. The study was approved by the institutional review board of Yokohama City University (No. A110929001). All 7 patients underwent VATS and the partially resected lung specimens were fixed with 10% buffered formalin and representative tissue sections were embedded in paraffin. Hematoxylin and eosin (HE) staining was performed for routine histological diagnosis. Neoplasm histologies were determined by 2 expert pulmonary pathologists (Y. N. and K. O.).

### DNA isolation and determination of *FLCN* germline mutations

DNA from peripheral blood leukocytes was obtained using the LabPass Blood Mini kit (Cosmo genetech, Seoul, South Korea) according to the manufacturer’s instructions. In 2 patients, blood samples were not available and DNA was extracted from normal lung tissue using the QIAamp DNA Mini kit (Qiagen GmbH, Hilden, Germany). Exons 4–14 of *FLCN* were amplified by PCR using the primers described previously [4]. PCR conditions were described in our previous study [18]. After purification, DNA was labeled with a Big Dye Terminator v1.1 Cycle Sequencing Kit (Life Technologies, Carlsbad, CA) and DNA sequencing was done using an ABI Prism 3100 Genetic Analyzer (Life Technologies).

### Somatic mutation analysis of *FLCN*, *EGFR* and *KRAS* in lung neoplasms

To detect possible somatic mutations of *FLCN*, *EGFR*, and *KRAS* in the lung neoplasms, DNA sequencing was done using the protocol described above. Tumor cells fixed in formalin and embedded in paraffin were selectively microdissected using an LMD 6500 (Leica Microsystems, Tokyo, Japan), and tumor DNA was extracted using the QIAamp DNA Mini kit (Qiagen). Hot spots including *KRAS* exons 2 and 3 and *EGFR* exons 19 and 21 were amplified for detection of heterozygous mutations. The following primers were used for *KRAS* and *EGFR*: *KRAS* exon 2, (F) 5’-ACATGTTCTAATATAGTCAC-3’ and (R) 5’-CAACAATAGAGGTAAATCTTGT -3’; *KRAS* exon 3, (F) 5’-TTCCTACAGGAAGCAAGTAG-3’ and (R) 5’-TGGGGA GGGCTTTCTTTGTG-3’; *EGFR* exon 19, (F) 5’-GCAATATCAGCCTTAGGT GCGGCTC-3’ and (R) 5’-CATAGAAAGTGAACATTTAGGATGTG-3’; amd *EGFR* exon 21, (F) 5’-CTAACGTTCGCCAGCCATAAGTCC-3’ and (R) 5’- GCTGCGAGCTCACCCAGAATGTCTGG-3’. The same *FLCN* primers were used as in germline mutation analysis. If only one of the alleles was amplified and the other allele was unreadable in the genetically mutated exon, it was determined that LOH occurred as a second hit [19].

### Immunohistochemistry and fluorescence *in situ* hybridization (FISH)

Four-μm-thick paraffin sections were subjected to immunohistochemistry and fluorescence *in situ* hybridization (FISH). After deparaffinization and rehydration, sections were autoclaved at 121°C for 15 min, after which they were treated with diluted antibodies at 4°C overnight. For immunostaining, the following reagents were used: rabbit polyclonal antibodies against phospho-mTOR (p-mTOR) (Ser2448) (Cell Signaling Technology, Danvers, MA), phospho-S6 ribosomal protein (p-S6) (Ser235/236) (Cell Signaling Technology), phospho-Akt (p-Akt) (Ser473) (Cell Signaling Technology), and FLCN (ab93196, Abcam, Cambridge, UK); rabbit monoclonal mutant/deleted-specific antibody against EGFR (L858R and E746-A750 deletion) (Cell Signaling Technology); rabbit monoclonal antibody against ROS1 (clone D4D6, Cell Signaling Technology); and mouse monoclonal antibody against ALK (clone 5A4, Abcam). Antibodies specific for TTF-1, Napsin-A, and Ki-67 were obtained from DAKO (Carpinteria, CA). Immunohistochemistry was performed using an ENVISION kit (DAKO) and the autoclave antigen retrieval technique according to the manufacturer’s protocol. Working dilutions were 1:250 for the ALK antibody and 1:100 for other antibodies. The intensity of immunostaining was semi-quantitatively graded according to the criteria used in our previous studies [19, 20]. The H-score method was used for ROS1 staining: an H-score of ≥ 150 indicated rearrangement of ROS1 [21]. To confirm the absence of *ROS1* rearrangement, FISH assays were also performed using a custom *ROS1* break-apart probe set according to the manufacturer’s protocol (GSP Laboratory, Kawasaki, Japan).

## Results

### Clinical findings and germline mutations

Seven patients (LP1–7) underwent VATS for resection of lung neoplasms. Surgically obtained tissues included both ground glass opacity (GGO) lesions and BHD-associated pulmonary cysts. One patient (LP1) had two independent lesions, and another (LP5) had seven independent lesions. Each of the other patients had a single lesion. Clinical findings and prognoses are summarized in Table 1. Five patients were never-smokers and the other two were current smokers. All patients had multiple pulmonary cysts, and five had episodes of pneumothorax. One patient (LP7) had undergone dialysis for 10 years for chronic renal failure, and was later diagnosed with dialysis-associated RCC and underwent bilateral nephrectomy. Another patient (LP6) had undergone surgery for thyroid cancer 8 years previously, and no recurrence was detected. The patient with a MPH-like lesion (LP4) had poorly differentiated gastric adenocarcinoma. In all patients, thorough medical examination and histological analyses excluded the possibility of metastatic lung cancer. MPH is known to develop as multiple small nodules in patients with tuberous sclerosis complex (TSC) [22]. Patient LP4, however, had neither symptoms nor a family history associated with TSC. One patient (LP2) died of lung cancer, and the others were alive without recurrent pulmonary neoplasms.

**Table 1:** Summary of clinical information.

Patient’s No.	Sex	Age	No. of lung neoplasm	Findings	Smoking	Prognosis (Months)
LP-1(proband)	Female	65	2	Skin papules, PC	Smoker	NED (12)
LP-2 (brother)	Male	59	1	PTX, PC	Smoker	Dead (16)
LP-3	Female	62	1	PTX, PC	Never	NED (38)
LP-4	Female	71	1	PTX, PC, Gastric cancer.	Never	NED (10)
LP-5	Female	54	7	PTX, PC	Never	NED (85)
LP-6	Female	72	1	Thyroid cancer, PC	Never	NED (10)
LP-7	Male	68	1	Skin papules, PTX, PC, RCCs	Never	NED (21)

Abbreviation: PC, pulmonary cysts; PTX, pneumothorax; NED, no evidence of disease; RCCs, renal cell carcinomas.

Four different patterns of *FLCN* germline mutations were identified. All patients exhibited frameshift mutations. Cytosine duplication in the C_8_ tract in the exon 11 (c.1285dupC) was the most frequent pattern; it was detected in 3 patients. A 7-bp duplication in exon 12 (12c.1347_1353 dupCCACCCT) was detected in 2 patients. The other patients demonstrated different mutation patterns (Table 2).

**Table 2:** Summary of histology, gene mutations and immunostaining.

Tumor No.	Histology	Size (mm)	Localization	Mutation				Immunostaining			
				*FLCN* (Germline)	*FLCN* (Somatic)	*EGFR*	*KRAS*	EGFR L858R	ROS1 H-score ^a^	p-mTOR and p-S6	Ki-67 labeling index (%)
LP1-T1	PAC	22X15	BV bundle	Exon 11c.1285dupC	11 LOH	L858R	W.T.	(+)	100 ^b^	(++)	5
LP1-T2	AAH	2x2	BV bundle		N.D.	N.D.	N.D.	(-)	(-)	(++)	3
LP2-T1	MPAC	Unknown	Unknown	Exon 11c.1285dupC	11 LOH	W.T.	G12D	(+)	(-)	(++)	70
LP3-T1	AIS	14x12	BV bundle	Exon 11c.1285dupC	Undetectable	W.T.	W.T.	(-)	(-)	(++)	4
LP4-T1	MPH	3x3	ILS	Exon 9c.906dupT	9 LOH	W.T.	W.T.	(-)	(-)	(+)	1
LP5-T1	AAH	3x3	BV bundle	Exon 12c.1347_1353 dupCCACCCT	N.D.	N.D.	N.D.	(-)	(-)	(++)	3
LP5-T2	AAH	2x2	BV bundle		N.D.	N.D.	N.D.	(-)	(-)	(++)	1
LP5-T3	AAH	3x2	BV bundle		N.D.	N.D.	N.D.	(-)	(-)	(++)	1
LP5-T4	AAH	3x3	BV bundle, ILS		N.D.	N.D.	N.D.	(-)	50 ^b^	(++)	4
LP5-T5	AAH	3x2	BV bundle, ILS		N.D.	N.D.	N.D.	(-)	100 ^b^	(++)	3
LP5-T6	AAH	3x2	BV bundle, ILS		N.D.	N.D.	N.D.	(-)	0	(++)	3
LP5-T7	AAH	4x3	ILS		N.D.	N.D.	N.D.	(-)	100 ^b^	(++)	3
LP6-T1	AIS	6x6	BV bundle	Exon 12c.1347_1353 dupCCACCCT	12 LOH	W.T.	W.T.	(-)	0	(++)	1
LP7-T1	MIA	14x3	BV bundle	7c.769_771delTCC	7 LOH	L858R	W.T.	(-)	0	(++)	5

Abbreviation: PAC, papillary adenocarcinoma; AAH, atypical alveolar hyperplasia; MPAC, micropapillary adenocarcinoma; AIS, adenocarcinoma *in situ*; MPH, micronodular pneumocyte hyperplasia; MIA, minimally invasive adenocarcinoma; BV, bronchovascular; ILS, interlobular septa; LOH, loss of heterozygosity; W.T., wild-type; N.D., not done;

^a^H-core is according to the reference 21;

^b^*ROS1* rearrangement was lacked by FISH analysis.

### Histological findings of lung neoplasms

**Fig 1:**
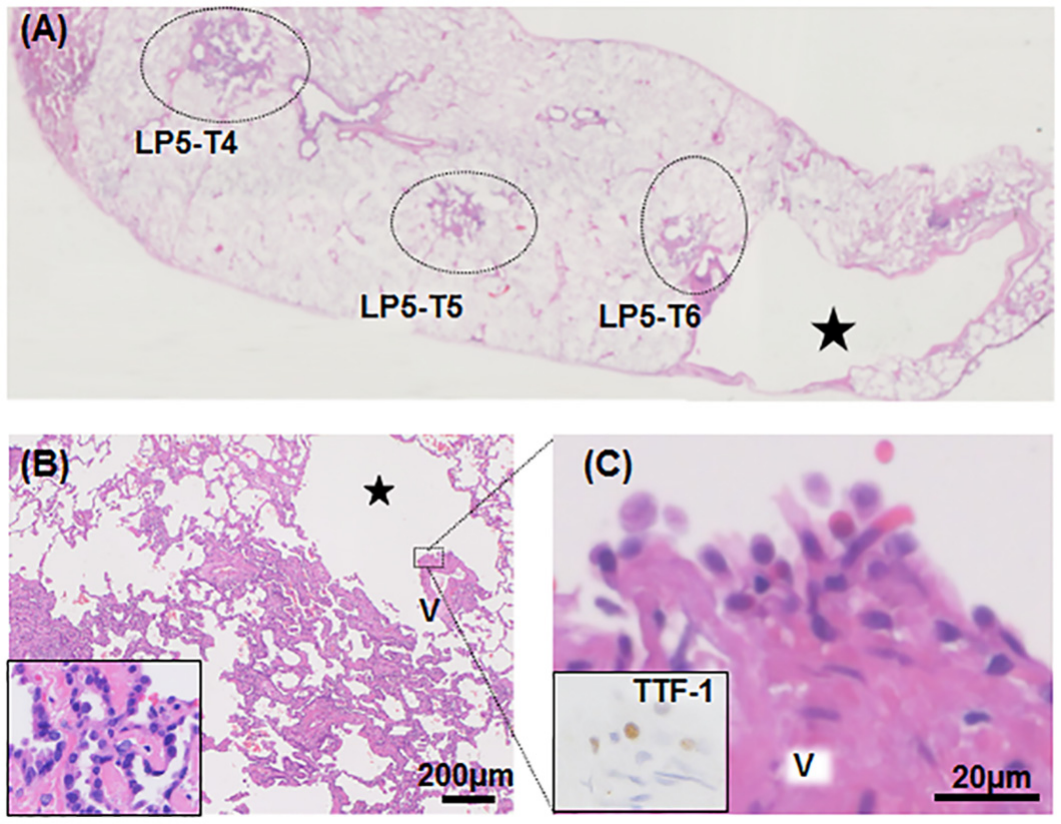
Histological features of atypical adenomatous hyperplasias (AAHs), adenocarcinoma *in situ* (AIS), and minimally invasive adenocarcinoma (MIA). Hematoxylin and eosin staining of resected lungs. BHD-associated cysts are indicated by stars. **(A)** Three AAH lesions independently developed in LP5. Each lesion is indicated by a dotted circle. **(B)** Higher magnification of an AAH lesion in LP5 is shown. This lesion is incorporated into a small cyst. V indicates a blood vessel. The higher magnification of the vein surrounded by a dotted box is shown in (C). Inset: Further magnification of the lesion. **(C)** Outer surface of the protruding vessel is lined by a layer of pneumocytes with mild atypia. V indicates a blood vessel. Inset: lining cells are immunostained for TTF-1.

The 14 lesions were subjected to detailed histological typing. Five lesions were diagnosed as adenocarcinomas, including two adenocarcinoma *in situ* (AIS). Eight noninvasive neoplasms were diagnosed as AAH. The histology of the nodular lesion in LP4 is described below. Almost all neoplasms developed in contact with the interlobular septa and/or bronchovascular bundle. AAHs, AISs and an MIA often include arranged cystic alveolar spaces and/or were fused to a cyst (Fig 1A and 1B). BHD pulmonary cysts often had small veins protruding into the cystic space. The surface of the protruding vein was sometimes lined by neoplastic cells (Fig 1B and 1C).

**Fig 2:**
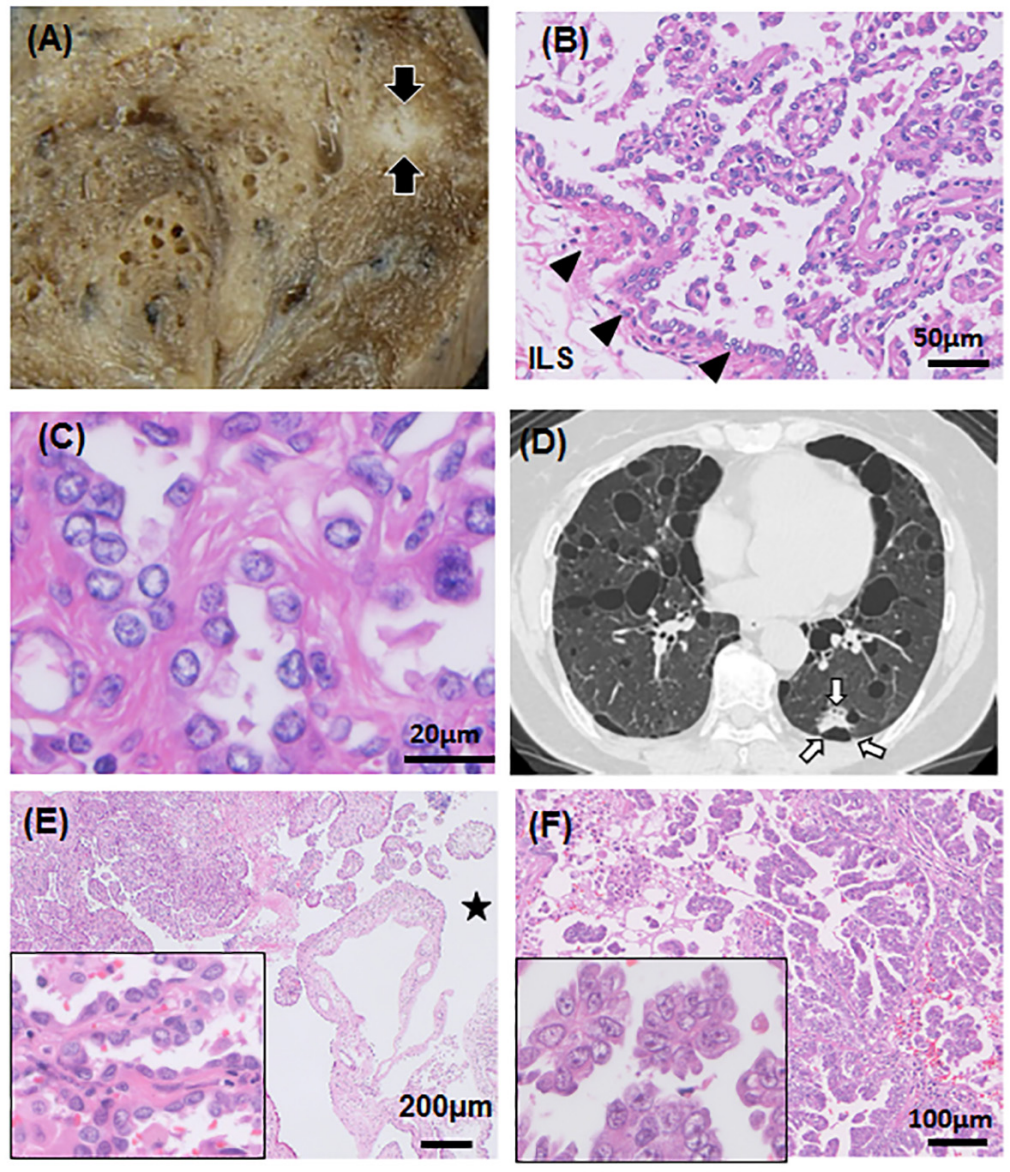
Histological features of a micronodular pneumocyte hyperplasia (MPH)-like lesion and adenocarcinomas. **(A)** The sectioned surface of LP4-T1 is shown. The arrows indicate the white nodule that corresponds to an MPH-like lesion. **(B)** Hematoxylin and eosin (HE) staining of the MPH-like lesion. The lesion borders on the interlobular septum (ILS), indicated by arrowheads. **(C)** Further magnification of the lesion. Plump pneumocytes have enlarged nuclei that lack overt atypia and mitosis. The alveolar septa are thickened with dense fibers. **(D)** Computed tomography of LP1 demonstrates multiple cysts and a ground-glass opacity lesion indicated by arrows. **(E)** HE staining of the papillary adenocarcinoma (LP1-T1). A star indicates the cyst infiltrated by cancer cells. Inset: Higher magnification of the lesion. **(F)** HE staining of the micropapillary adenocarcinoma (LP2-T1). Inset: Higher magnification of the lesion.

In the micronodular lesion obtained from LP4, the proliferating cells had plump nuclei and the cells lacked overt atypia of adenocarcinoma (Fig 2A–2C). Mitosis was not evident, and the Ki-67 labeling index was 1%. The nodule was well-demarcated and had a papillary configuration rather than the typical lepidic pattern of AAH. Alveolar septa were thickened with dense fibrous mesenchyme. The lesion had histological characteristics similar to those of MPH that are occasionally observed in TSC lung. The presence of lymphangiomyomatosis (LAM) cells was not detected in the patient’s lung.

Familial invasive adenocarcinomas were detected in LP1 and LP 2, who were siblings with a smoking history [15]. The papillary adenocarcinoma (PAC) occurring in the proband (LP1-T1) was located in between a few cystic spaces (Fig 2D). The lesion exhibited a papillary structure with a fibrovascular core, and it partially infiltrated adjacent to a subpleural cyst (Fig 2E). The micropapillary adenocarcinoma (MPAC) occurring in her brother (LP2-T1) showed a predominantly non-mucinous micropapillary pattern (Fig 2F).

### Activated mTOR signaling in neoplastic lesions

**Fig 3:**
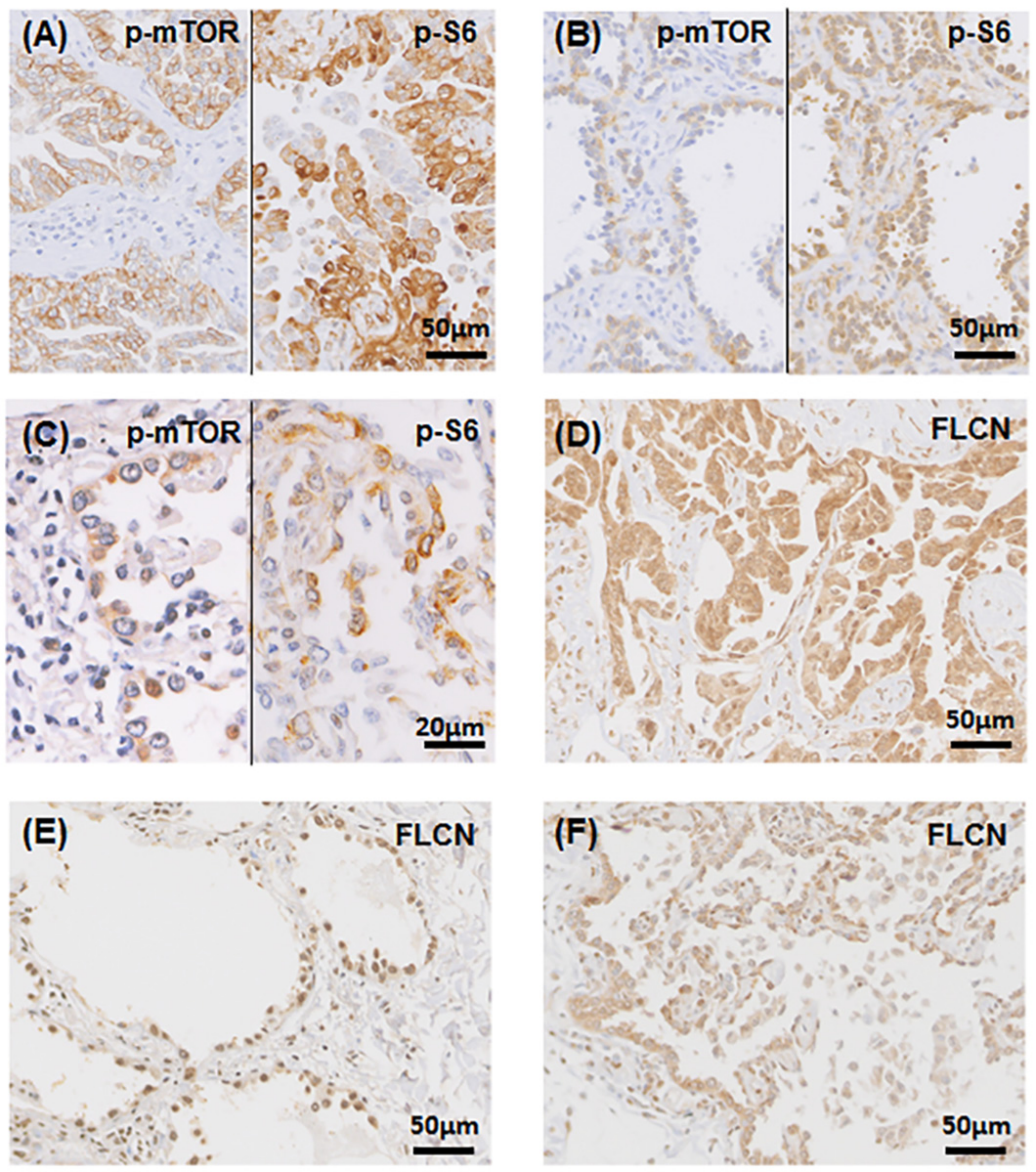
Expression of phospho-mTOR (p-mTOR), phospho-S6 (p-S6), and *FLCN* in BHD lung neoplasms. ** (A)-(C)** The micropapillary adenocarcinoma (MPAC) (A), adenocarcinoma *in situ* (AIS) (B), and micronodular pneumocyte hyperplasia (MPH)-like lesion (C) show positive immunostaining for p-mTOR (left) and p-S6 (right). Lower p-mTOR and p-S6 staining intensities are observed in the MPH-like lesion compared to the adenocarcinomas. **(D)-(F)** The MPAC (D), AIS (E), and MPH-like lesion (F) are diffusely immunostained for *FLCN*.

We investigated immunohistochemical expression of p-mTOR, p-S6, p-Akt and FLCN in neoplastic lesions. Neoplastic epithelial cells of all lesions demonstrated positive immunostaining for p-mTOR and p-S6 (Fig 3A–3C). In an MPH-like lesion, the proliferating cells showed weaker immunoreactivity for p-mTOR and p-S6 compared to other neoplastic lesions (Fig 3C). In normal-looking areas, the pulmonary epithelia were negative for p-mTOR and p-S6, as we previously reported [11]. None of the neoplasms were positively stained for p-Akt (data not shown). All neoplastic cells were positively stained for FLCN (Fig 3D–3F). The MPAC case showed the highest Ki-67 labeling index (70%), while the others had indices ranging from 1–5% positive cells (Table 2).

### Possible gene mutations (*FLCN*, *EGFR*, *KRAS*, *ALK*, and *ROS1*) in neoplastic lesions

**Fig 4:**
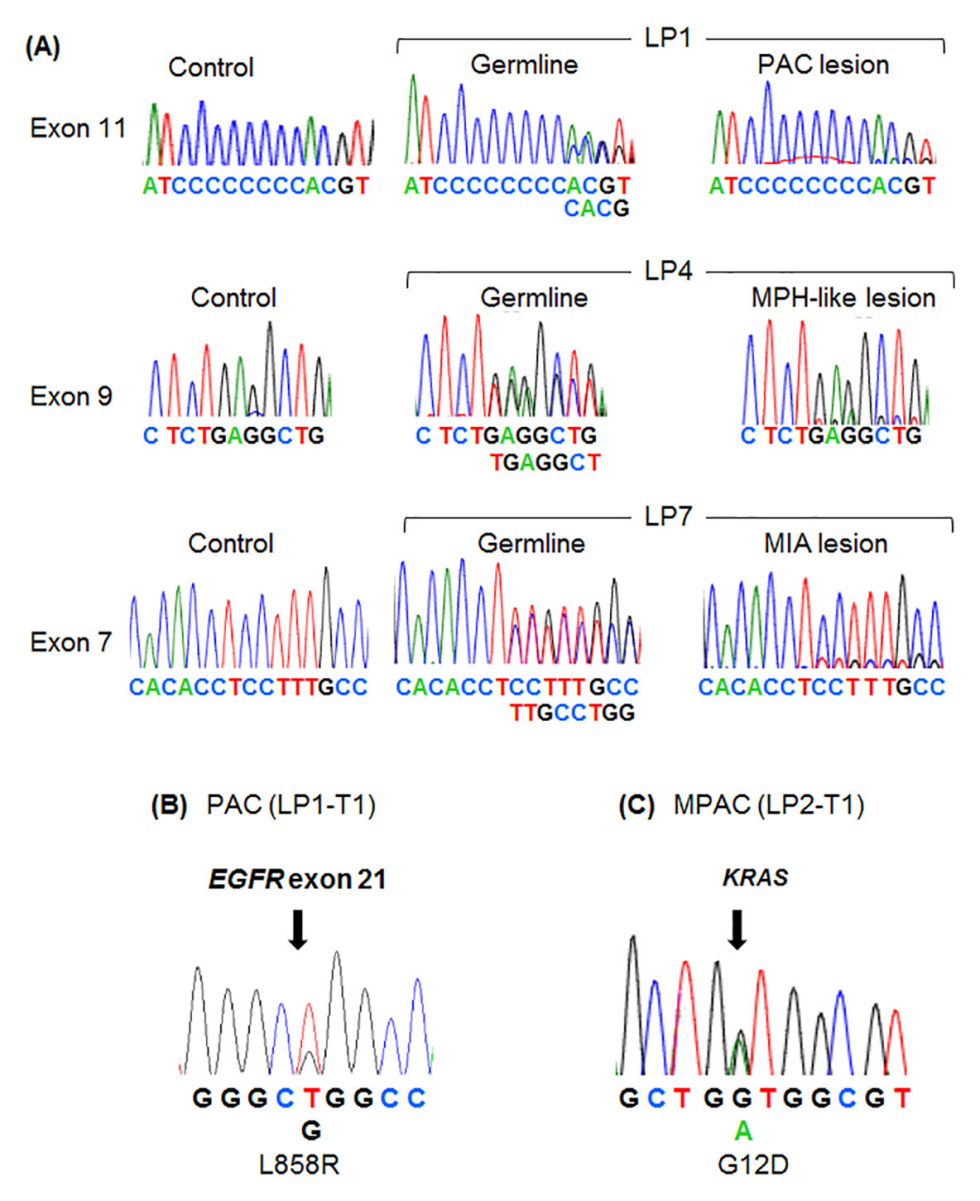
Sequence analysis of *FLCN*, *EGFR*, and *KRAS* in BHD lung neoplasms. ** (A)** Germline and somatic *FLCN* status in 3 representative cases with different mutation patterns are shown. Control normal sequences are shown on the left. Germline mutations are shown on the middle. The somatic status of *FLCN* in microdissected neoplasms are shown on the right. **(B)** The papillary adenocarcinoma (PAC) had a heterozygous missense mutation (L858R) in *EGFR* (indicated by an arrow). **(C)** The micropapillary adenocarcinoma (MPAC) had a heterozygous missense mutation (G12D) in *KRAS* (right, indicated by an arrow).

The somatic *FLCN* status was investigated in the five microdissected adenocarcinomas and the single MPH-like lesion. Five of these six tumors demonstrated hemizygous sequence patterns (Fig 4A, right panels). Therefore, the neoplasms were determined to have undergone LOH. Interestingly, all of these lesions were wild-type for *FLCN*. The AAH lesions were not available for second-hit analysis, because these smaller lesions (2–3 mm in diameter) did not yield sufficient DNA by laser-capture microdissection.

Key genes associated with sporadic lung carcinomas include mutant *EGFR*, *KRAS*, *ALK*, and *ROS1*. We investigated possible somatic events in these genes using sequence analysis, immunostaining, and FISH. Sequence analysis revealed a somatic mutation of *EGFR* exon 21 (L858R) in the MIA and PAC cases (Fig 4B). In all of the investigated lesions, *EGFR* exon 19 was wild-type. A somatic mutation of *KRAS* in codon 12 (G12D) was detected only in the MPAC case (Fig 4C). The other neoplasms exhibited wild-type *KRAS*. None of the neoplasms were immunostained for E746-A750 deletion-specific EGFR or ALK. Four neoplasms were weakly immunostained for ROS1, and two neoplasms were weakly immunostained for L858R-specific EGFR. ROS1 H-scores were ≤150. FISH analysis confirmed that none of the samples had *ROS1* rearrangement (data not shown).

## Discussion

The possibility of an increased risk of lung cancer in BHD patients has not been previously discussed. This report described for the first time histopathological features and somatic *FLCN* events in BHD lung neoplasms of adenocarcinomatous lineage using a substantial number of cases. In this study, 1 patient possessed multiple AAHs and another had two independent neoplasms, indicating that they might be prone to multifocal tumorigenesis. The neoplasms were often located close to the bronchovascular bundles and/or interlobular septa with which the cysts were incorporated. Since BHD pulmonary cysts are preferentially located in these regions [12, 18, 23], a neoplasm and a cyst might abut on a common interstitial niche. Contrary to the hybrid oncocytic/chromophobe tumor that is characteristic of BHD-associated RCCs, most of the lung cases looked like unremarkable glandular neoplasms indistinguishable from sporadic counterparts, except for 1 case, an MPH-like lesion.

One BHD patient (LP4) had an MPH-like lesion. Both FLCN and TSC are known to regulate mTOR signaling. The kidney, skin, and lung are commonly affected organs in BHD patients as well as those with TSC. Hayashi *et al*. reported that all eight MPH lesions occurring in patients with TSC had LOH either in *TSC1* or *TSC2* [22], suggesting that MPH is a neoplastic disorder. Although the current World Health Organization classification does not list MPH as a tumor, the present study supports the findings that MPH may be neoplastic. LP4 provided new etiologic information: MPH lesions appear to occur not only in TSC lung but also in BHD lung. TSC-associated MPH activates the mTOR pathway [22], and the MPH lesion of LP4 showed expression of activates mTOR pathway components. The staining intensities for p-mTOR and p-S6 were weaker compared to those in AAHs and adenocarcinomas, which might reflect less aggressive behavior.

The present somatic mutation analysis of *FLCN* revealed that five of the six adenocarcinoma/MPH-like neoplasms had LOH, and that the wild-type copy of *FLCN* was preserved in all lesions with LOH. These lung lesions demonstrated a nuclear immunostaining pattern for FLCN (Fig 3D–3F), indicating that biallelic deletion of *FLCN* is unlikely [11, 19]. BHD-associated RCCs with *FLCN* LOH lack the wild-type allele, and these RCCs show weak cytoplasmic staining for FLCN [19, 24]. Therefore, the somatic state of *FLCN* and the expression level of FLCN in lung neoplasms appear to be somewhat different from those in RCC. Unfortunately, further cytogenetic and protein analyses were not possible due to the fact that these were archived formalin-fixed, paraffin-embedded tissues. A subject for future study is whether wild-type FLCN is produced haploinsufficiently or if a nonspecific molecule cross-reacts with the antibody. In our previous study of RCCs, we detected monoallelic somatic mutation of *FLCN* in 1 patient [19]. Together, these findings indicate that BHD patients possibly bear mutant *FLCN*-associated neoplasms, while preserving the wild-type allele.

Not only invasive adenocarcinomas but also noninvasive neoplasms showed *FLCN* LOH, which indicates that the somatic event alone may not be sufficient for pulmonary neoplasms to transform to an aggressive phenotype. BHD-associated RCCs grow slowly in most cases, and have balanced chromosomes even in the cases with *FLCN* LOH in which the wild-type allele is deleted, because these segments exist as unipatrental disomy [25]. Human lung cancers potentially demonstrate synergistic mutations/deletions of more than one cancer-related genes, such as *LKB1* deletion with *KRAS* mutation, in a subset of poorly differentiated adenocarcinomas [26]. It is tempting to hypothesize that LOH of *FLCN*, concomitant with oncogenic mutations in *EGFR* or *KRAS*, might allow low-grade lung neoplasms to progress to invasive adenocarcinomas.

Lung cancer is one of the most frequent malignancies. Therefore, the present data should be interpreted with caution regarding whether the patients with germline *FLCN* mutations have an increased risk of developing lung cancer compared to individuals without this genetic disorder. We have collected clinical information from 150 individuals with BHD diagnosed by genetic testing. Although we could not obtain full family medical histories, the morbidity of patients with lung neoplasms was 4.67%. Histological findings have suggested that genetically fragile *FLCN* might contribute to focally aberrant proliferation of pulmonary epithelia, albeit at a low incidence. It is currently unknown whether synergism of *EGFR*/*KRAS* and *FLCN* LOH accelerates tumor progression in the lung. We hope that this study of lung neoplasms that occurred in 7 patients with BHD will be informative for both physicians and BHD patients and result in better medical care. While BHD patients are recommended to undergo periodic RCC screening, there is no clinical guideline for lung screening. The present study may prompt physicians to consider periodic follow-up of BHD lungs. Further laboratory and clinical studies are required to clarify this issue. An additional subject of future study is whether somatic mutation or deletion of *FLCN* is observed in a subset of sporadic lung neoplasms.
